# Immunogenicity of immunomodulatory, antibody-based, oncology therapeutics

**DOI:** 10.1186/s40425-019-0586-0

**Published:** 2019-04-15

**Authors:** Jasmine Davda, Paul Declerck, Siwen Hu-Lieskovan, Timothy P. Hickling, Ira A. Jacobs, Jeffrey Chou, Shahram Salek-Ardakani, Eugenia Kraynov

**Affiliations:** 1 Pfizer, San Diego, CA USA; 2 University of Leuven, Leuven, Belgium; 3 David Geffen School of Medicine at UCLA, Los Angeles, CA USA; 4 Pfizer, Andover, MA USA; 5 Pfizer, 219 East 42nd Street, New York, NY 10017-5755 USA; 6 Pfizer, San Francisco, CA USA

**Keywords:** ADA, Antibody, Immunogenicity, Immunomodulatory, Neutralizing antibodies, PD-1/PD-L1, CTLA-4

## Abstract

The increasing use of multiple immunomodulatory (IMD) agents for cancer therapies (e.g. antibodies targeting immune checkpoints, bispecific antibodies, and chimeric antigen receptor [CAR]-T cells), is raising questions on their potential immunogenicity and effects on treatment. In this review, we outline the mechanisms of action (MOA) of approved, antibody-based IMD agents, potentially related to their immunogenicity, and discuss the reported incidence of anti-drug antibodies (ADA) as well as their clinical relevance in patients with cancer. In addition, we discuss the impact of the administration route and potential strategies to reduce the incidence of ADA and manage treated patients. Analysis of published reports indicated that the risk of immunogenicity did not appear to correlate with the MOA of anti-programmed death 1 (PD-1)/PD-ligand 1 monoclonal antibodies nor to substantially affect treatment with most of these agents in the majority of patients evaluated to date. Treatment with B-cell depleting agents appears associated with a low risk of immunogenicity. No significant difference in ADA incidence was found between the intravenous and subcutaneous administration routes for a panel of non-oncology IMD antibodies. Additionally, while the data suggest a higher likelihood of immunogenicity for antibodies with T-cell or antigen-presenting cell (APC) targets versus B-cell targets, it is possible to have targets expressed on APCs or T cells and still have a low incidence of immunogenicity.

**Electronic supplementary material:**

The online version of this article (10.1186/s40425-019-0586-0) contains supplementary material, which is available to authorized users.

## Introduction

In the past few years, immune checkpoint inhibitors, such as the anti-cytotoxic T-lymphocyte antigen 4 (CTLA-4) monoclonal antibody (mAb) ipilimumab, the anti-programmed death 1 (PD-1) mAbs nivolumab, pembrolizumab, and cemiplimab, and the anti-PD-ligand 1 (PD-L1) mAbs atezolizumab, avelumab, and durvalumab, have revolutionized treatment regimens for several malignancies (Table 1) [1–7]. Other immuno-oncology mAbs are currently being evaluated in clinical trials, including novel anti-CTLA-4 and anti-PD-1 mAbs, and antibodies that target costimulatory receptors in the tumor necrosis factor receptor (TNFR) superfamily (i.e. CD137/4-1BB, OX40, CD40, GITR, and CD27), TIM-3, LAG-3, and other receptors [8–15]. Further, approved or investigational agents with different structures/composition and immunomodulatory (IMD) activities (i.e. bispecific antibodies, chimeric antigen receptor (CAR)-T cells, antibody fragments, antibody-drug conjugates [ADC], and fusion proteins) are also being administered to patients [16–19].

**Table 1:** Immune checkpoint inhibitor mAb therapies

mAb	Target	Structure	Isotype	Route of admin.	Approvals^a^
Ipilimumab	CTLA-4	Fully human	IgG1, kappa	IV	MEL, RCC^b^, MSI-H/MRD CRC^b^
Nivolumab	PD-1	Fully human	IgG4, kappa	IV	MEL, NSCLC, cHL, RCC, HNSCC, UC, MSI-H/MRD CRC, HCC
Pembrolizumab	PD-1	Humanized	IgG4, kappa	IV	MEL, NSCLC, cHL, HNSCC, PMBCL, UC, MSI-H/MRD cancer, HCC, gastric cancer, cervical cancer
Cemiplimab	PD-1	Fully human	IgG4, kappa	IV	CSCC
Atezolizumab	PD-L1	Humanized Fc-engineered	IgG1, kappa	IV	UC, NSCLC
Avelumab	PD-L1	Fully human	IgG1, lambda	IV	MCC, UC
Durvalumab	PD-L1	Engineered human	IgG1, kappa	IV	UC, stage III NSCLC

*CRC* colorectal cancer, *cHL* classical Hodgkin lymphoma, *CSCC* cutaneous squamous-cell carcinoma, *CTLA-4* cytotoxic T-lymphocyte associated protein 4, *HCC* hepatocellular carcinoma, *HNSCC* head and neck squamous cell carcinoma, *IV* intravenous, *mAb* monoclonal antibody, *MCC* Merkel cell carcinoma, *MEL* melanoma, *MRD* mismatch repair-deficient, *MSI-H* microsatellite instability-high, *NSCLC* non-small-cell lung cancer, *PD-1* programmed death 1, *PD-L1* PD-ligand 1, *PMBCL* primary mediastinal large B-cell lymphoma, *RCC* renal cell carcinoma, *SC* subcutaneous, *UC* urothelial carcinoma

^a^Listed approvals reflect US Food and Drug Administration approvals

^b^In combination with nivolumab

Thus, the expected increase in the use of multiple IMD agents in the same patient, for sequential or simultaneous combination therapies, has raised questions about their potential immunogenicity and related effects on the safety and efficacy of treatment compared with non-IMD agents [20–22]. Distinct from B-cell depleting antibodies (i.e. anti-CD20 or anti-CD19 mAbs), other IMD agents have the capacity to strengthen host immune responses, including both antitumor and autoimmune responses in some cases [23, 24]. Consequently, they might directly, or indirectly through stimulation of immune networks, affect humoral immune responses resulting in the induction of anti-drug antibodies (ADA). ADA can induce infusion-related reactions or alter the pharmacokinetics (PK) of an agent by affecting its clearance [25]. In addition, in some cases ADA can decrease treatment efficacy by neutralizing the activity of the drug (neutralizing antibody, NAb). Parameters usually reported for ADA include incidence (i.e. percentage of positive patients), titers, and evolution over time (i.e. present or absent at baseline, emergent during treatment or boosted by treatment, transient or persistent) [21, 22].

Furthermore, the subcutaneous (SC) route of administration is being increasingly explored clinically for the delivery of IMD agents to provide more convenience to patients (e.g. anti-PD1 or PD-L1 antibodies SC in trials NCT03656718, NCT03665597, NCT02573259, NCT02827968, and NCT03735121 or blinatumomab SC in NCT02961881). This raises questions on their potential immunogenicity, compared with intravenous (IV) delivery of these agents [26–30]. Proteins administered SC may be taken up and processed by dendritic cells (DCs), which function as primary antigen-presenting cells (APCs), more easily than IV agents, with initiation of specific immune responses [26, 27].

To address some of these topics, we review the mechanisms of action (MOA) of approved, antibody-based, IMD agents potentially related to their immunogenicity and discuss the evidence reported to date on the incidence of ADA and NAb across multiple, approved IMD and non-IMD agents and the clinical relevance for cancer patients. In addition, we discuss potential strategies to reduce the incidence of immunogenicity and manage treated patients.

### The immune system and antibody-based IMD agents

IMD agents may potentiate immune responses through direct immunomodulation (e.g. anti-CTLA-4 or anti-PD-1/PD-L1 mAbs) or they may inhibit immune responses, as in the case of proapoptotic/depleting antibodies that target immune cells (e.g. anti-CD19 or anti-CD20 antibodies). IMD agents developed to induce antitumor immune responses in cancer patients exert immunostimulatory activity through different MOAs. For example, in lymphoid tissues, T-cell activation triggers surface expression of the immune checkpoint CTLA-4, which binds to B7 molecules with stronger affinity than CD28, blocking the costimulation signal. Consequently, CTLA-4 inhibition by ipilimumab can result in costimulation and T-cell activation and contribute to more effective antitumor responses [1, 31].

PD-1 expression by tumor-infiltrating lymphocytes is associated with impaired function and decreased survival, proliferation, cytokine release, and cytotoxicity against tumor cells. Inhibition of this tumor-associated immunosuppressive pathway by agents targeted to PD-1 or its ligand PD-L1, was shown to restore functional antitumor immune responses in experimental tumor models and in treated patients [2, 3, 31]. In addition, combined inhibition of the PD-1 and CTLA-4 pathways may yield synergistic effects, suggesting that they are both critically involved in regulating T-cell activity [32, 33].

More complex antibody-based agents can induce targeted activation of immune cells. Bispecific antibodies are recombinant molecules engineered to express two binding specificities and allow concomitant engagement of a tumor-associated antigen and immune cells [16]. Depending on their structure, they may be small, bivalent molecules with variable regions connected by a linker, such as the anti-CD3/CD19 bispecific T-cell engager (BiTE) blinatumomab, or more complex antibody-based modalities such as tetravalent antibodies, in which a single-chain variable fragment (scFv) with one antigen specificity is fused to the c-terminal of an IgG with a different antigen specificity, resulting in tetravalent molecules with two binding sites for each antigen. Significant clinical activity has been observed with blinatumomab, approved for the treatment of patients with B-cell precursor acute lymphoblastic leukemia (ALL), in the presence of autologous T cells [16, 34].

CAR-T cells are engineered T cells expressing receptors that bind target antigens using an antibody-derived scFv, which allows T cells to bypass the restrictions imposed by the major histocompatibility complex (MHC). Autologous CAR-T cells derived from the patient are expected to be less immunogenic and potentially more persistent in vivo than allogeneic CAR-T cells derived from healthy donors. Addition of costimulatory regions (i.e. CD28, CD137/4-1BB) to second- and third-generation CARs has also contributed to increased in vivo T-cell persistence and expansion. Although treatment with CAR-T cells may be associated with cytokine-release syndrome in a substantial number of patients, durable responses have been achieved with CD19-targeted CAR-T cells in patients with B-cell ALL and non-Hodgkin lymphoma (NHL) [17].

### Immunogenicity testing of antibody-based, anti-cancer agents

Multiple factors may affect the immunogenicity of a mAb, either related to the mAb itself (i.e. product-related impurities, excipients, dosing regimen) or the patient (i.e. type and stage of disease, potential comorbidities, prior and concomitant treatments). In addition, assay-related variables (i.e. sensitivity and specificity, drug concentration in test samples, and cut-off points in confirmatory assays), may affect ADA and NAb measurements [21, 22]. Bioanalytical approaches for the detection of ADA and NAb have been described in detail in other reviews [35–37].

Developing fully human or humanized antibodies lowers the risk of inducing or boosting ADA compared with murine/human chimeric mAbs, and this approach has been used for all approved immune checkpoint inhibitor antibodies targeting PD-1, PD-L1, and CTLA-4 **(**Table 1**)** [38–50]. However, patients may still develop ADA/Nab able to affect treatment with these antibodies [21]. To date, immunogenicity has been evaluated in large numbers of patients for approved mAbs (Table 2), while more limited or no information is available for mAbs or combinations still at the investigational stage. All mAbs included in this analysis were administered intravenously. When specified, ADA were assessed by electrochemiluminescence (ECL) assays, with the exception of an ipilimumab study that relied on a bead-based assay [48]. Information available on the extent of drug interference in ADA evaluations is included in the footnotes of Table 2.

**Table 2:** Incidence of anti-drug antibodies (ADA) and neutralizing antibodies (NAb) reported in patients treated with immune checkpoint inhibitor mAbs^a^

mAb	Patients *N*, regimen, tumor type	ADA assay	ADA %	NAb %	Reference	Year
Anti-PD-1						
Nivolumab	*N* = 1086, pool of 6 clinical studies	ECL	12.7	0.8	Agrawal et al.	2017
	*N* = 2085	ECL	11.2	0.7	US PI	2018
	Nivo 3 mg/kg followed by ipi 1 mg/kg Q3W	NR	23.8-26 ADA to nivo	0.5–1.9 nivo NAb	US PI	2018
	Nivo 1 mg/kg followed by ipi 3 mg/kg Q3W	NR	37.8 ADA to nivo	4.6 nivo NAb	US PI	2018
	Nivo followed by ipi	NR	4.1–8.4 ADA to ipi	0–0.3 ipi NAb	US PI	2018
Pembrolizumab	*N* = 1087	ECL	1.7	NR	Van Vugt et al.	2016
	NSCLC	ECL	2.5	NR	Van Vugt et al.	2016
	melanoma	ECL	0.7	NR	Van Vugt et al.	2016
	*N* = 1289^b^	ECL	2.1	0.5	US PI	2018
Cemiplimab	*N* = 398	ECL	1.3	NR	US PI	2018
Anti-CTLA-4						
Ipilimumab	*N* = 1024, melanoma	ECL	1.1	0	US PI	2018
	*N* = 144, melanoma^c^	ECL	4.9	0	US PI	2018
	Nivo and ipi, *N* = 499, RCC and mCRC	NR	5.4 ADA to ipi	0 ipi NAb	US PI	2018
	*N* = 31, melanoma	Bead-based assay	26	NR	Knerveland et al.	2018
Anti-PD-L1						
Atezolizumab	*N* = 2007	NR	39.1	NR	EMA	2017
	*N* = 111 (cohort 1), urothelial carcinoma	NR	48	NR	US PI	2018
	*N* = 275 (cohort 2), urothelial carcinoma	NR	42	NR	US PI	2018
	*N* = 565, NSCLC	NR.	30	NR	US PI	2018
Avelumab	*N* = 1558	NR	4.1	NR	US PI	2017
	*N* = 1738^d^	NR	5.9	NR	EMA	2017
Durvalumab	*N* = 1570	NR	2.9	NR	US PI	2018
	Durvalumab + tremelimumab, *N* = 60	ECL	6.6 ADA to durva	NR	Antonia et al	2016
	Durvalumab + tremelimumab, *N* = 53	ECL	1.8 ADA to trem	NR	Antonia et al	2016

*ADA* anti-drug antibody, *durva* durvalumab, *CTLA-4* cytotoxic T-lymphocyte associated protein 4, *ECL* electrochemiluminescent bridging assay, *EMA* European Medicines Agency, *ipi* ipilimumab, *mAb* monoclonal antibody, *mCRC* metastatic colorectal cancer, *NHL* non-Hodgkin lymphoma, *NAb* neutralizing antibody, *nivo* nivolumab, *NR* not reported, *NSCLC* non-small-cell lung cancer, *PD-1* programmed death 1, *PD-L1* PD-ligand 1, *Q2W* every 2 weeks, *Q3W* every 3 weeks, *RCC* renal cell carcinoma, *trem* tremelimumab, *US PI* United States product information

^a^All mAbs were administered intravenously

^b^Analysis performed in patient samples with concentrations below the drug tolerance level of the ADA assay

^c^ADA assessed using an ECL assay with improved drug tolerance

^d^Assay performed with a cut point known to provide adequate assay sensitivity and drug tolerance level

#### Anti-PD-1/PDL-1 and anti-CTLA-4 antibodies

For immune checkpoint inhibitors, a low incidence of ADA (0–12.7%) has been reported following single-agent treatment with the anti-PD-1 mAbs nivolumab, pembrolizumab, and cemiplimab; the anti CTLA-4 mAb ipilimumab; and the anti-PD-L1 mAbs avelumab and durvalumab in patients with advanced malignancies (Table 2) [38–47]. NAb were detected in 0–0.8% of patients treated with nivolumab, pembrolizumab, or ipilimumab monotherapy [39, 41, 42]. No clinically relevant effects on safety, PK, or efficacy were noted following development of ADA/NAb to nivolumab, pembrolizumab, or ipilimumab, when analyzed in large patient populations enrolled in prospective trials [38–43]. Differently from previous analyses, findings with ipilimumab from a small, observational study in patients with metastatic melanoma suggested that ADA may affect treatment outcomes [48]. Eight (26%) patients were ADA-positive at any time point and the presence of ADA significantly correlated with shorter median overall survival (mOS; 235 vs 658 days, *p* = 0.03). The differences in ADA incidence reported for ipilimumab may reflect the use of assays with different detection thresholds, requiring further validation in larger, prospective studies [48].

Among patients evaluable for ADA against atezolizumab, a humanized, Fc-engineered, anti-PD-L1 IgG1 mAb, 39.1% of patients (pooled from multiple clinical trials) were reported to have post-treatment ADA (Table 2). Hypersensitivity reactions occurred in 1.2% of patients. In advanced urothelial carcinoma, treatment-emergent ADA were detected at ≥1 time point in 42–48% of patients and found not to affect safety of treatment. However, observed systemic exposures for atezolizumab were lower in ADA-positive patients due to increased clearance [49, 50]. In advanced non-small-cell lung cancer (NSCLC), 30% of evaluated patients tested positive for treatment-emergent ADA at ≥1 time point. Median time to ADA formation was 3 weeks. Similar to the observation in patients with urothelial carcinoma, ADA-positive patients with NSCLC had decreased atezolizumab exposure. Furthermore, in an exploratory analysis, ADA-positive patients (21%) appeared to derive less benefit compared with ADA-negative patients, with a mOS similar to that observed in the docetaxel control arm (9.6 months) [50]. No clinically relevant effects were noted on incidence and severity of adverse events (AEs) in patients positive for anti-atezolizumab antibodies [49, 50].

Varying effects on ADA have been observed when combining an anti-PD-1/PD-L1 and an anti-CTLA-4 mAb. Higher incidences of ADA (23.8–37.8%) and NAb (0.5–4.6%) were observed against nivolumab but not against ipilimumab, following administration of nivolumab and ipilimumab to patients with advanced solid tumors (Table 2) [39]. However, no effects on the PK profile of nivolumab or an increase in infusion-related reactions were reported in the patients that developed anti-nivolumab antibodies. Preliminary results from a small phase I, combination study of durvalumab and the anti-CTLA-4 mAb tremelimumab indicated low levels of ADA against durvalumab (6.6%) and tremelimumab (1.8%) following treatment in patients with advanced NSCLC. No apparent correlations were observed between the presence of ADA and treatment tolerability or antitumor activity [51].

#### Antibody-based IMD agents for hematologic malignancies

Drugs used to target differentiation antigens expressed on both malignant and normal hematologic cells, particularly B cells and their progenitors, might be expected to decrease their own immunogenicity through immunomodulation as well. ADA have been reported in < 1% of patients with relapsed/refractory CD19^+^ B-cell precursor ALL treated with the anti-CD3/CD19 BiTE blinatumomab (~ 54 kDa) [52]. Treatment with the anti-CD22 ADC inotuzumab ozogamicin in patients with relapsed/refractory CD22^+^ B-cell precursor ALL was also associated with a low incidence of ADA (3%), with no effect on the clearance of inotuzumab ozogamicin in ADA-positive patients [53]. Infusion-related reactions were reported in 2% of patients receiving inotuzumab ozogamicin, generally occurring at the end of infusion in cycle 1 [53]. Similarly, treatment with anti-CD20 mAbs (ofatumumab, obinutuzumab, and rituximab) for B cell malignancies as well as the anti-CD52 mAb alemtuzumab for B-cell chronic lymphocytic leukemia or the anti-CD38 mAb daratumumab for multiple myeloma resulted in no to low incidences of ADA development, with no observed ADA-related effects on treatment safety and efficacy [21, 54, 55]. Infusion reactions were reported in 40% of patients at first infusion of daratumumab and in 2–4% of patients at subsequent infusions [55].

Seven percent of patients treated with the anti-CD30 ADC brentuximab vedotin, used to treat Hodgkin and other types of lymphoma, developed persistent ADA and 30% of patients had a transient ADA response to the drug [56, 57]. The effects of anti-brentuximab vedotin antibodies on treatment efficacy are not known. Overall, 10% of patients receiving brentuximab vedotin monotherapy experienced infusion-related reactions; 1% of patients with persistently positive ADA developed infusion reactions resulting in treatment discontinuation [57].

For CAR-T cells, most of the patients (86–91.4%) receiving anti-CD19 genetically-modified, autologous T cells tisagenlecleucel (indicated for relapsed/refractory B-cell ALL and diffuse large B-cell lymphoma [DLBCL]), were positive for anti-murine CAR19 antibodies prior to infusion [58, 59]. Treatment-induced, anti-murine CAR19 antibodies were detected in 5% of the patients. Pre-existing and treatment-induced anti-mCAR19 antibodies were reported not to affect expansion/persistence of tisagenlecleucel, safety, or clinical response to treatment [58]. More limited data are currently available for the anti-CD19 genetically-modified, autologous T cells axicabtagene ciloleucel (indicated for adult patients with relapsed/refractory DLBCL). Three (2.8%) patients were reported positive at baseline and on study for antibodies against FMC63 (the originating molecule for this CAR), with no observed effects on treatment [60].

### Incidence of ADA with IMD versus non-IMD agents

In a larger analysis, we systematically reviewed package inserts and/or journal articles summarizing clinical ADA incidences reported for 40 IMD and 19 non-IMD agents in oncological or non-oncological indications (references provided in Additional file 1: Tables S1 and S2). In this broader context, IMD agents include all drugs that may directly or indirectly modulate (inhibit or potentiate) immune cells. The analysis suggested that 8% of the oncology IMD mAbs, 22% of the non-oncology IMD mAbs, and 11% of the non-IMD mAbs were associated with higher ADA incidence rates (≥15%). The incidence of ADA for the combined (oncology and non-oncology) IMD mAbs did not appear significantly different from that of non-IMD mAbs (0–83% vs 0–27%; Wilcoxon rank-sum test, *p* = 0.4). However, the likelihood of high immunogenicity appeared to be greater for IMD mAbs (18% of the IMD vs 11% of the non-IMD agents evaluated had high ADA). In general, B cell-depleting mAbs were associated with low incidences of ADA (< 15%) as would be expected from their mechanism of action.

No significant difference in ADA incidence was observed between human (0–53%) and humanized (0–83%) IMD mAbs (*p* = 0.9). High ADA incidence rates were reported for 24%, 4%, and 27% of agents with targets expressed on myeloid APCs (DCs, macrophages, and monocytes combined), B cells, and T cells, respectively. These findings suggest a lower likelihood of ADA with agents targeted to B cells than T cells or myeloid APCs. However, it is possible to have targets expressed on APCs or T cells and still have a low incidence of ADA (e.g. ipilimumab, nivolumab, pembrolizumab).

Evaluation of the impact of the administration route on immunogenicity was limited by the lack of approved oncology IMD biologics administered SC as they are still at the investigational stage. However, analysis of the ADA incidence for 16 non-oncology IMD agents administered SC (Additional file 1: Table S1) showed that the majority (with the exception of adalimumab, golimumab, daclizumab, and ixekizumab) were associated with an ADA incidence < 15%, consistent with previous findings [28]. No significant difference in ADA incidence was observed between mAbs administered IV (0–83%) or SC (< 0.1–53%) in this data set (Wilcoxon rank-sum test, *p* = 0.2).

### Prediction of immunogenicity risk

While analysis of clinical ADA data for approved drugs suggests a relatively low risk of immunogenicity for many IMD agents, it is important to note that compounds that show high ADA incidences during clinical development typically do not progress to become drugs and this information may not be available in the public domain. Therefore, it is possible that the overall incidence of immunogenicity for IMD agents may be higher than reported in the literature. This highlights the need for predictive assays that can be applied preclinically to identify the IMD compounds with high potential for triggering immunogenicity in patients.

During preclinical development, both in silico algorithms and in vitro assays can be used to help select molecules with lower immunogenicity risk [61]. In silico algorithms to predict potential T cell epitopes are commonly used [62]. Confirmation of predicted sequences binding to MHC molecules can be obtained by in vitro binding assays [63]. Peptide sequences can be screened in peripheral blood mononuclear cell assays, enabling the design or selection of low-risk sequences to be included in the final candidate molecule. Assessment of immunogenicity signals for a protein product can be made with a DC-activation assay [64]. Uptake and processing of therapeutic proteins by DCs can be assessed with MHC-associated peptide proteomics (MAPPs) [65] to identify presented peptides, or the uptake and trafficking can be evaluated with optical methods 64]. Additional quantitative data on the number of T cells that might recognize a therapeutic protein can be obtained through restimulation experiments [66]. At present, in vitro B-cell assays for immunogenicity risk assessment have not been described.

Integrating readouts from various in silico and in vitro assays is not intuitive and provides only qualitative risk assessments [67]. A system for synthesis of data for quantitative assessment of risk and impact of immunogenicity is being investigated with a prototype mathematical model that uses in vitro assay data to simulate trial outcomes in terms of ADA incidence and impact on PK [68].

### Mitigating and managing antibody immunogenicity

A number of strategies have been proposed to limit the immunogenicity of antibody-based therapeutics and manage patients prior and during treatment with these agents.

Beyond the use of fully human/humanized mAbs, which is already widely implemented owing to technological advances in antibody development, selection of appropriate dosing regimens and administration schedules for each agent may further reduce the risk of inducing or boosting ADA.

Removal of T-cell and B-cell epitopes from biologic agents through protein engineering may contribute to mitigate their immunogenicity by limiting B-cell activation. Such a ‘deimmunization’ process has been investigated for CD22- and CD25-targeted, cytotoxic immunotoxins, with evidence of reduced antigenicity and immunogenicity in experimental models and in patients with hairy-cell leukemia [69, 70]. A further reduction in the immunogenic potential of fully human/humanized antibodies may be achieved in the future by designing novel molecules that contain determinants able to induce specific tolerance in a patient’s immune system. Tolerization processes may include the use of antibodies engineered to express regions able to stimulate T-regulatory cells with inhibitory activity on humoral immune responses or combinations with tolerance-inducing nanoparticles [71, 72].

Nanobody-based biologics represent an additional approach being explored to reduce the potential for immunogenicity. Nanobodies are cloned antibody fragments that contain only the variable regions of heavy-chain-only antibodies from camelideae. Thus, they are substantially smaller (i.e. 15 kDa) and potentially less immunogenic than conventional antibody molecules [73, 74].

In the clinic, premedication with antihistamines, acetaminophen, and/or corticosteroids, as currently applied, may help to prevent infusion-related reactions [75, 76]. Timely diagnoses and prompt therapeutic interventions may further limit the seriousness of hypersensitivity and infusion-related reactions observed in patients receiving biologic agents [75]. During administration, mild to moderate reactions can be managed in most patients by the temporary suspension of the infusion, a reduction in the infusion rate, and symptom management. In case of more severe reactions, treatment should be discontinued [75].

Corticosteroids and other immunosuppressive agents (i.e. methotrexate) have proven effective in reducing immunogenicity in patients with autoimmunity treated with mAbs, thus allowing long-term antibody administration [77–81]. Availability of more data in the future may provide further insight into the effectiveness of immunosuppressive approaches for the management of ADA-related reactions that may occur following administration of IMD agents in cancer patients. Selective B-cell depletion induced by anti-CD20 antibodies may contribute to further reduce humoral immune responses and thus ADA responses, either as a single agent or in combination regimens. Consistent with this hypothesis, patients with follicular lymphoma and other NHLs receiving combination treatment with rituximab and the anti-4-1BB/CD137 mAb utomilumab had a substantially lower incidence of treatment-emergent ADA against utomilumab compared with monotherapy [13, 15].

## Conclusions

The availability of multiple IMD agents, with comparable MOAs and different structures or routes of administration, may provide useful, alternative modalities for tailored treatment of cancer patients.

Overall, there is a general consensus that comparisons of the incidence and characteristics of ADA directed against an antibody-based agent should be interpreted with caution if the ADA were measured in different laboratories. Even more so, comparisons of immunogenicity findings among different agents, assessed in different studies and in heterogeneous patient populations, are limited by the variability of the measurements involved in each of these analyses [29]. Ultimately, potential effects of ADA and NAb on safety, PK, pharmacodynamics, and consequently overall efficacy are expected to provide the most relevant information for clinicians selecting specific treatment options for their cancer patients.

Nonetheless, the current experience with anti-PD-1/PD-L1 mAbs indicates that, while immunogenicity may require further investigations and appropriate management in some cases, the risk of ADA responses does not appear to correlate with the MOA nor to substantially affect the PK profile, safety, or efficacy of treatment with most of these agents in the majority of patients evaluated to date. Treatment with B-cell depleting agents appears associated with a low risk of immunogenicity.

Results from our analyses showed no significant difference in ADA incidence between IV and SC administration routes for non-oncology, IMD antibodies. Furthermore, while the data suggest a higher likelihood of ADA for antibodies with T-cell or APC targets versus B-cell targets, it is possible to have a target expressed on APCs or T cells and still have a low incidence of ADA. As more SC IMD oncology antibodies are evaluated clinically, further insights should be gained on whether SC administration influences the risk of immunogenicity in this class of agents.

The benefit demonstrated in terms of long-term responses and disease control by approved IMD therapies underscores the importance of effectively implementing these treatment strategies. Since, in daily practice, cancer patients may present a broader heterogeneity in characteristics, prior treatments, and comorbidities compared with the selected populations included in clinical trials, consideration of potential ADA responses may provide, in some cases, additional insight while selecting appropriate treatment modalities for each patient.

## Additional file

Additional file 1: **Table S1.** Immunomodulatory agents. **Table S2.** Non-immunomodulatory agents. (PDF 317 kb)

## Abbreviations

ADA: Anti-drug antibody
ADC: Antibody-drug conjugate
AE: Adverse event
ALL: Acute lymphoblastic leukemia
APC: Antigen-presenting cells
BiTE: Bispecific T-cell engager
CAR: Chimeric antigen receptor
CTLA-4: Cytotoxic T-lymphocyte antigen 4
DC: Dendritic cell
DLBCL: Diffuse large B-cell lymphoma
ECL: Electrochemiluminescence
EGFR: Epidermal growth factor receptor
EpCAM: Epithelial cell adhesion molecule
IMD: Immunomodulatory
IV: Intravenous
mAb: monoclonal antibody
MAPP: MHC-associated peptide proteomics
MHC: Major histocompatibility complex
MOA: Mechanism of action
mOS: median overall survival
Nab: Neutralizing antibody
NHL: Non-Hodgkin lymphoma
NSCLC: Non-small-cell lung cancer
PD-1: Programmed death-1
PD-L1: PD-ligand 1
PK: Pharmacokinetics
SC: Subcutaneous
scFv: single-chain variable fragment
TNFR: Tumor necrosis factor receptor
VEGF: Vascular endothelial growth factor
