# Supplementary material for: Immunogenicity of immunomodulatory, antibody-based, oncology therapeutics
Source: J Immunother Cancer. 2019 Apr 15;7:105. doi: 10.1186/s40425-019-0586-0 (PMC6466770; doi:10.1186/s40425-019-0586-0)
Supplement: Supplementary file 1 — Table S1. Immunomodulatory agents. Table S2. Non-immunomodulatory agents. (PDF 317 kb) [file 40425_2019_586_MOESM1_ESM.pdf]

Davda et al. SUPPLEMENTARY INFORMATION

Supplementary Table S1 Immunomodulatory agents

Agent	Target	Target cell types	Structure	ROA	Patient population from which ADA data are listed	Immunogenicity (% treated patients) ADA	NAb	Reference
**Oncology Agents**								
Alemtuzumab	CD52	Lymphocytes, monocytes, DCs	IgG1, humanized	IV	B-cell chronic lymphocytic leukemia	8.3	2	Package insert
Atezolizumab	PDL-1	Monocytes/macrophages, DCs, activated T cells, keratinocytes, placental trophoblasts, myocardial endothelium, cortical thymic epithelial cells	IgG1, aglycosylated, humanized	IV	Lung and bladder cancer	30-48	NR	Package insert
Avelumab	PD-L1	Monocytes/macrophages, DCs, activated T cells, keratinocytes, placental trophoblasts, myocardial endothelium, cortical thymic epithelial cells	IgG1, human	IV	Merkel cell carcinoma	4.1	NR	Package insert
Blinatumomab	CD19 / CD3	B cells, follicular DCs	BiTE	IV	Acute lymphoblastic leukemia	1	<1	Package insert
Brentuximab vedotin	CD30	Surface of sALCL cells and Hodgkin Reed-Sternberg (HRS) cells in classical HL. Limited expression on healthy tissue	IgG1, chimeric; ADC with MMAE payload	IV	Hodgkin lymphoma, systemic anaplastic large-cell lymphoma	7	4.2	Package insert
Cantuzumab mertansine	CanAg epitope on MUC1	Activated T cells, B cells, epithelial cells	IgG1, humanized; ADC with DM1 payload	IV	Colorectal cancer	0	NR	Tolcher et al. J. Clin. Oncol. 2003; 21:211–22
Gemtuzumab ozogamicin	CD33	Monocytes, granulocytes, myeloblasts, promyelocytes, myelocytes, metamyelocytes	IgG4, humanized, ADC with calicheamicin payload	IV	Acute myeloid leukemia	1.1	NR	Hock et al. AAPS J. 2015; 17:35–43

Ipilimumab	CTLA-4	T cells, activated B cells	IgG1, human	IV	Metastatic melanoma	4.9-5.4	0	Package insert
Nivolumab	PD-1	Subsets of thymocytes, activated T cells, B cells, myeloid cells	IgG4, human	IV	Melanoma, non-small cell lung cancer	8.5	0.6	Package insert
Obinutuzumab	CD20	Pre-B-cells, resting and activated B cells, follicular DCs, a small subset of circulating T lymphocytes	IgG1, humanized; afucosylated	IV	Chronic lymphocytic leukemia	0.2-7	NR	Package insert
Ofatumumab	CD20	Pre-B-cells, resting and activated B cells, follicular DCs, a small subset of circulating T lymphocytes	IgG1, human	IV	Chronic lymphocytic leukemia	<1	NR	Package insert
Pembrolizumab	PD-1	Subsets of thymocytes, activated T cells, B cells, myeloid cells	IgG4, humanized	IV	Melanoma	2.1	0	Package insert
Tremelimumab	CTLA-4	T cells, activated B cells, DCs	IgG2, human	IV	Melanoma, solid tumors	0	NR	Millward et al. Br J Cancer. 2013; 108:1998–2004
**Non-oncology Agents**								
Adalimumab	TNF	T cells, monocytes, macrophages, neutrophils, endothelial cells, hepatocytes, chondrocytes, fibroblasts	IgG1, human	SC	Rheumatoid arthritis	53	76	Cohen et al. Annals Rheum. Dis. 2018; 77:914-921
Alemtuzumab	CD52	Lymphocytes, monocytes, DCs	IgG1, humanized	IV	Multiple sclerosis	29-83	78	Package insert
Basiliximab	IL-2Rα	Subsets of thymocytes and peripheral blood lymphocytes including CD4+CD25+ regulatory T cells and memory T cells, activated B cells, macrophages	IgG1, chimeric	IV	Renal transplant	1.2-1.5	NR	Package insert
Belimumab	BLyS (BAFF)	B cells, some populations of myeloid cells, DCs, bone marrow stromal cells	IgG1, human	IV/SC	Systemic lupus erythematosus	4.8	0.5	Package insert
Brodalumab	IL-17Rα	Fibroblasts, epithelial and endothelial cells, monocytes/macrophages, mast cells (broad expression)	IgG2, human	SC	Psoriasis	3	0	Package insert

Canakinumab	IL-1β	B cells, neutrophils, monocytes, bone marrow progenitor cells	IgG1, human	SC	CAPS, SJIA	1.5-3.1	0	Package insert
Certolizumab pegol	TNF	T cells, monocytes, macrophages, neutrophils, endothelial cells, hepatocytes, chondrocytes, fibroblasts	Humanized Fab, PEGylated	SC	Crohn's disease	7-8	6	Package insert
Daclizumab	IL-2R	Subsets of thymocytes and peripheral blood lymphocytes including CD4+CD25+ regulatory T cells and memory T cells	IgG1, humanized	SC	Multiple sclerosis	19	8	Package insert
Denosumab	RANK-L	T- and B-cells, osteoclasts	IgG2, human	SC	Bone loss	<1	0	Package insert
Dupilumab	IL-4Rα	B and T cells, and a variety of other hematopoietic/non-hematopoietic cells	IgG4, human	SC	Atopic dermatitis	7	1	Package insert
Eculizumab	C5	DCs, mast cells, macrophages, B cells, T cells	IgG2/4, humanized	IV	Paroxysmal nocturnal hemoglobinuria	3-5	0	Package insert
Efalizumab	CD11a	Leukocytes	IgG1, humanized	SC	Psoriasis	6.3	N.R.	Package insert
Golimumab	TNF	T cells, monocytes, macrophages, neutrophils, endothelial cells, hepatocytes, chondrocytes, fibroblasts	IgG1, human	SC	Rheumatoid arthritis, PsA	16-28	4	Package insert
Infliximab	TNF	T cells, monocytes, macrophages, neutrophils, endothelial cells, hepatocytes, chondrocytes, fibroblasts	IgG1, chimeric	IV	Crohn's disease	10-51	NR	Package insert
Ixekizumab	IL-17α	Fibroblasts, epithelial and endothelial cells, monocytes/macrophages, mast cells (broad expression)	IgG4, humanized	SC	Psoriasis	9-22	2	Package insert
Mepolizumab	IL-5	B1 B cells, eosinophils, basophils, mast cells	IgG1, humanized	SC	Severe eosinophilic asthma	6	0	Package insert
Natalizumab	α4 integrin	Lymphocytes, monocytes, thymocytes, NK cells, some B and T cells	IgG4, humanized	IV	Multiple sclerosis	6-10	6	Package insert
Ocrelizumab	CD20	Pre-B cells, resting and activated B cells, follicular DCs, a small subset of	IgG1, humanized	IV	Multiple sclerosis	1	0.2	Package insert

		circulating T lymphocytes						
Omalizumab	IgE	B cells, monocytes, macrophages, eosinophils, platelets, DCs, Langerhans cells	IgG1, humanized	SC	Asthma	<0.1	0	Package insert
Reslizumab	IL-5	B1 B cells, eosinophils, basophils, mast cells	IgG4, humanized	IV	Severe asthma	4.8-5.4	NR	Package insert
Rituximab	CD20	Pre-B-cells, resting and activated B cells, follicular DCs, a small subset of circulating T lymphocytes	IgG1, chimeric	IV	Rheumatoid arthritis	11	NR	Package insert
Sarilumab	IL-6R	Activated plasma cells, most leucocytes, epithelial cells, fibroblasts, hepatocytes, skeletal muscle and neural cells	IgG1, human	SC	Rheumatoid arthritis	9.2	6.7	Package insert
Secukinumab	IL-17α	Fibroblasts, epithelial and endothelial cells, monocytes/macrophages and mast cells (broad expression)	IgG1, human	SC	Psoriasis	<1	0.2	Package insert
Siltuximab	IL-6	Activated plasma cells, most leucocytes, epithelial cells, fibroblasts, hepatocytes, skeletal muscle and neural cells	IgG1, chimeric	IV	Castleman disease	0.2	0	Package insert
Tocilizumab	IL-6R	Activated plasma cells, most leucocytes, epithelial cells, fibroblasts, hepatocytes, skeletal muscle and neural cells	IgG1, humanized	IV	Rheumatoid arthritis	2	1.1	Package insert
Ustekinumab	P40 subunit of IL-12 and 23	Macrophages, DCs, monocytes, Langerhans cells, neutrophils, keratinocytes	IgG1, human	IV/SC	Psoriasis, Crohn's disease	6-12.4	6	Package insert
Vedolizumab	α4β7 integrin	Most mature lymphocytes, small subsets of thymic and bone marrow cells	IgG1, humanized	IV	Ulcerative colitis, Crohn's disease	4-13	0.6	Package insert

ADA anti-drug antibody, *ADC* antibody-drug conjugate, *BiTE* bi-specific T-cell engager, *CAPS* cryopyrin-associated periodic syndromes, *CD* Crohn's disease, *CTLA-4* cytotoxic T-lymphocyte–associated antigen 4, *DC* dendritic cell, *DM1* emtansine, *IL* interleukin, *IV* intravenous, *MMAE* monomethyl auristatin E, *NAb* neutralizing antibody, *NR* not reported, *PD-1* programmed death 1, *PD-L1* PD-ligand 1, *PsA* psoriatic arthritis, *R* receptor, *ROA* route of administration, *SC* subcutaneous, *SJIA* Still's juvenile idiopathic arthritis, *TNF* tumor necrosis factor

Supplementary Table S2 Non-immunomodulatory agents

Agent	Target	Structure	Immunogenicity (% treated patients)	Reference
			ADA	
Abciximab	GPIIb/IIIa	Fab, chimeric	6-27	Package insert
Dinutuximab	GD2	IgG1, chimeric	13-18	Package insert
Alirocumab	PCSK9	IgG1, human	4.8	Package insert
Guselkumab	IL-23	IgG1, human	6.0	Package insert
Ramucirumab	VEGF-R2	IgG1, human	3.0	Package insert
Ado-trastuzumab emtansine	HER2	IgG1, humanized, ADC	5.3	Package insert
Cetuximab	EGFR	IgG1, chimeric	5.0	Package insert
Ranibizumab	VEGF	Fab, humanized	1-9	Package insert
Panitumumab	EGFR	IgG2, human	4.6	Package insert
Necitumumab	EGFR	IgG1, human	4.1	Package insert
Idarucizumab	Dabigatran	Fab, humanized	4.0	Package insert
Olaratumab	PDGFRα	IgG1, human	3.5	Package insert
Pertuzumab	HER2	IgG1, humanized	2.8	Package insert
Palivizumab	RSV	IgG1, humanized	1.1-1.5	Package insert
Bevacizumab	VEGF	IgG1, humanized	0.6	Package insert
Evolocumab	PCSK9	IgG2, human	0.1	Package insert
Trastuzumab	HER2	IgG1, humanized	0.1	Package insert
Bezlotoxumab	*C. difficile* toxin B	IgG, human	0	Package insert
Raxibacumab	Anthrax	IgG1, human	0	Package insert

ADA anti-drug antibody, *ADC* antibody-drug conjugate, *EGFR* epithelial growth factor receptor, *GD2* disialoganglioside 2, *IL-23* interleukin 23, *PDGFRα* platelet-derived growth factor receptor α, *PCSK9*, proprotein convertase subtilisin/kexin type 9, *RSV* respiratory syncytial virus, *VEGF* vascular endothelial growth factor, *VEGF-R2* VEGF-receptor 2
